# Fabrication and characterization of GaN-based light-emitting diodes without pre-activation of p-type GaN

**DOI:** 10.1186/s11671-015-0792-8

**Published:** 2015-02-27

**Authors:** Xiao-Long Hu, Hong Wang, Xi-Chun Zhang

**Affiliations:** Engineering Research Center for Optoelectronics of Guangdong Province, School of Physics and Optoelectronics, South China University of Technology, Guangzhou, Guangdong 510640 China

**Keywords:** p-type GaN, Light-emitting diodes, Indium tin oxide, Hall effect, 81.05.Ea, 85.60.Bt, 73.40.Cg

## Abstract

We fabricated GaN-based light-emitting diodes (LEDs) without pre-activation of p-type GaN. During the fabrication process, a 100-nm-thick indium tin oxide film was served as the p-type contact layer and annealed at 500°C in N_2_ ambient for 20 min to increase its transparency as well as to activate the p-type GaN. The electrical measurements showed that the LEDs were featured by a lower forward voltage and higher wall-plug efficiency in comparison with LEDs using pre-activation of p-type GaN. We discussed the mechanism of activation of p-type GaN at 500°C in N_2_ ambient. Furthermore, x-ray photoemission spectroscopy examinations were carried out to study the improved electrical performances of the LEDs without pre-activation of p-type GaN.

## Background

GaN-based materials are of great importance to fabricate visible and ultraviolet light-emitting diodes (LEDs) [1-3]. In order to improve the performances of the LEDs, highly conductive p-type GaN and reliable ohmic contacts are required. However, the as-grown p-type GaN is of high resistance due to the Mg-H complexes [4-6]. In order to dissociate the Mg-H complexes, thermal annealing at above 700°C in N_2_ ambient is usually employed [7,8]. Nevertheless, such high temperature would deteriorate the crystalline quality of the InGaN active layer [9-11]. Recently, it was reported that thermal annealing in O_2_ ambient was an alternative way for activation of p-type GaN [12]. This is mainly attributed to the decrease of hydrogen concentration during the reaction of oxygen with hydrogen [8-13]. On the other side, reliable p-type ohmic contacts with high optical transmittance are technologically important for realizing high-performance GaN-based LEDs. Ni/Au contact is usually used as semitransparent ohmic electrodes as well as current spreading layer for GaN-based LEDs [14,15]. However, the light transmittance of the Ni/Au contact is relatively low. Indium tin oxide (ITO) contact layer is considered to be a good candidate for transparent contacts because of its high transparency and high conductivity [14]. However, due to its relatively small work function (4.8 eV), the ITO film that directly deposited on p-type GaN would yield a leaky Schottky contact [16]. Thus, schemes employing intermediate layers such as NiO, short-period-superlattice, or InGaN strained contact layer structure have been proposed to obtain a good ohmic contact between ITO and p-type GaN [16-18]. So far, the activation of p-type GaN and the realization of ohmic contact proceeded separately, which made the device fabrication process relatively complicated. More importantly, high temperature or long-time annealing heat treatments would further exacerbate the degradation of the device performances. In this paper, we fabricated GaN-based LEDs without pre-activation of p-type GaN. During the fabrication process, the devices were annealed only once at 500°C in N_2_ ambient for 20 min to increase the transparency of ITO contact layer as well as to activate the p-type GaN. The electrical measurements showed that the devices were featured by a lower forward voltage and higher wall-plug efficiency (WPE) in comparison with LEDs pre-annealed either at 800°C in N_2_ ambient or at 500°C in O_2_ ambient. The mechanisms for improvement of LED performances will be discussed in detail in this study.

## Methods

The LED epitaxial samples studied in this work were grown by a metal organic chemical vapor deposition (MOCVD) system on (0001)-oriented patterned sapphire substrate. The epitaxial structure was comprised of 3.5-μm-thick undoped GaN, 2.5-μm-thick n-type GaN, 0.2-μm-thick InGaN/GaN multiple-quantum-wells (MQWs) active region, 25-nm-thick AlGaN electron blocking layer, and 270-nm-thick p-type GaN layer, which capped with 2.0-nm-thick InGaN strained layer. For a comparative study, we prepared three LED epitaxial samples marked as sample A, sample B, and sample C. To activate the p-type GaN, sample A was pre-annealed at 800°C in N_2_ ambient for 20 min, while sample B was pre-annealed at 500°C in O_2_ ambient for 10 min. For sample C, no pre-annealing heat treatment was carried out. Photoluminescence (PL) spectra of the three samples were measured using a 405 nm semiconductor laser before the following LED device fabrication process. The fabrication process of the three samples proceeded as follows. Patterned 210-nm-thick SiO_2_ layer was first deposited on p-type GaN by plasma-enhanced chemical vapor deposition as a current blocking layer. A 100-nm-thick ITO film was then evaporated by an electron beam evaporator as a transparent conducting layer. Subsequently, a mesa with an area of 8 mil × 16 mil was defined by using standard photolithography and dry etching. The samples were annealed at 500°C in N_2_ ambient for 20 min to increase the transparency of the ITO layer, after which the transparency increased from 90% to 98% at the wavelength of 460 nm. Finally, patterned Cr/Pt/Au layer was deposited as n- and p-electrodes, and the LED samples with chip size of 9 mil × 17 mil were obtained. Current-voltage and light output power measurements were performed using the FitTech IPT6000 LED chip/wafer probing and testing system (Fittech Co., Ltd., Taichung, Taiwan).

## Results and discussion

**Figure 1:**
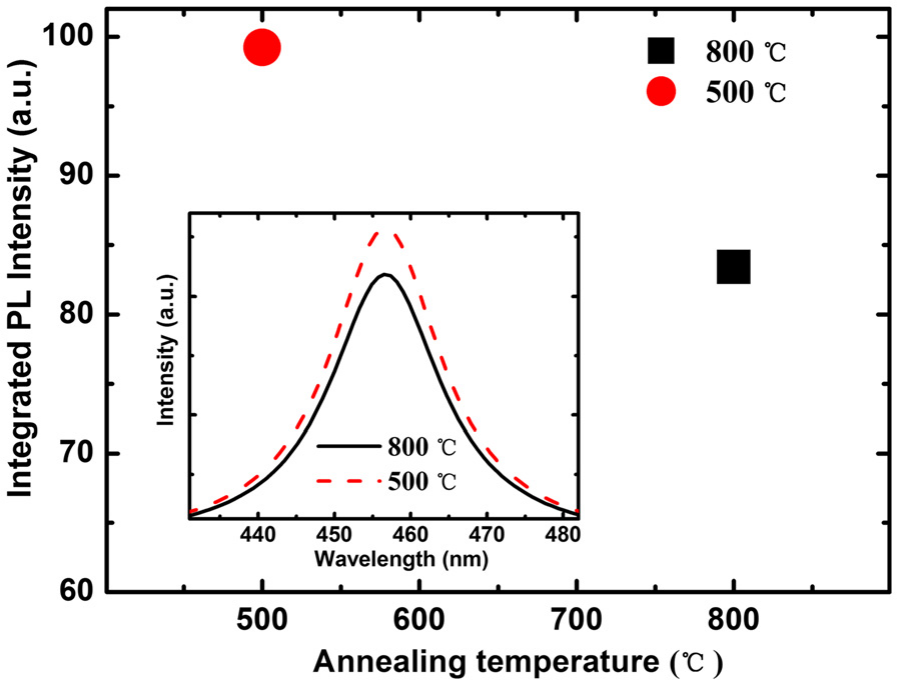
**Integrated PL intensity of two LED epitaxial wafers.** The inset shows the PL spectra of two wafers.

Figure 1 shows the integrated PL intensity of LED epitaxial wafers pre-annealed at 800°C in N_2_ ambient for 20 min and pre-annealed at 500°C in O_2_ ambient for 10 min. The inset shows the PL spectra of the two wafers. The integrated PL intensity of the wafers is the average value of 100 points measured from the entire 2-in. epitaxial layers. Assuming the integrated PL intensity of as-grown wafer is 100, the intensities of the wafers pre-annealed at 800°C and pre-annealed at 500°C are decreased to 83.4% and 99.2%, respectively. The obvious decrease in the integrated PL intensity of the wafer pre-annealed at 800°C is due to the increased non-radiative recombination that caused by the defects generated during the high-temperature thermal annealing process [11,19,20].

**Figure 2:**
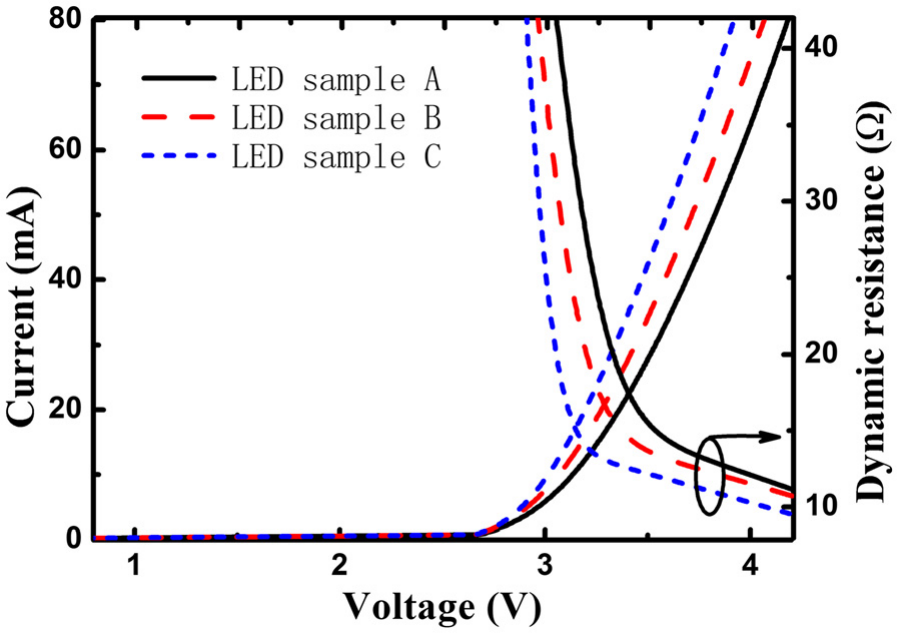
**Current-voltage and dynamic resistance characteristics of the LED samples A, B, and C, respectively.**

Figure 2 shows the current-voltage and dynamic resistance characteristics of the LED samples A, B, and C. The forward voltage is the average value of 100 LED chips which are uniformly distributed on the entire 2-in wafer, and the current-voltage curve was measured from the device of an average level. It is found that the forward voltages (dynamic resistances) of the LED samples A, B, and C are 3.36 V (18.5 Ω), 3.27 V (16.9 Ω), and 3.19 V (14.0 Ω) under an injection current of 20 mA, respectively. The forward voltage of the LED sample B is lower than that of the LED sample A. This could be due to the high activation efficiency of Mg dopant in the p-type GaN layer during the thermal annealing in O_2_ ambient [11]. The results also show that the forward voltage and dynamic resistance of the LED sample C without pre-activation of the p-type GaN are the lowest in these devices, which prove that the p-type GaN was effectively activated during the fabrication process. It is generally believed that the activation temperature of p-type GaN in N_2_ ambient should be higher than 700°C to dissociate the Mg-H complexes. However, in our circumstances, the p-type GaN in LED sample C was activated at a relatively low temperature of 500°C in N_2_ ambient. We consider that this is attributed to the covered ITO film. The mechanism of activation of p-type GaN at a relatively low temperature in N_2_ ambient will be discussed later.

**Figure 3:**
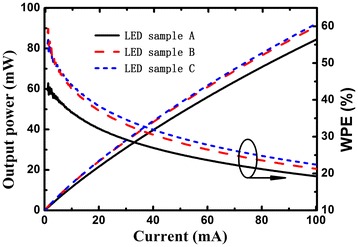
**Output power and WPE versus injection current for LED samples A, B, and C, respectively.**

The light output power as a function of injection current for LED samples A, B, and C is shown in Figure 3. It is found that the light output power of LED sample A (21.8 mW) is much lower than that of LED sample B (24.5 mW) and LED sample C (24.6 mW). We believe that the defects, which were generated during the high-temperature thermal annealing process, increased the non-radiative recombination and ultimately lead to a decrease of output power, and this is consist with the results in PL measurements. It is also shown that the WPE of LED samples A, B, and C is 32.0%, 36.9%, and 38.2% at an operating current of 20 mA, respectively. Therefore, the WPE of LED sample C is 19.4% higher than that of LED sample A. The decrease in the WPE for LED sample A is due to the lower light output power and the higher forward voltage. In addition, the WPE of LED sample C demonstrated a 3.5% improvement in comparison with that of LED sample B, which is mainly attributed to the lower forward voltage of LED sample C. The Hall effect and x-ray photoemission spectroscopy (XPS) measurements were carried out in the following experiment to explain the improved performance for LED sample C. In order to clarify the mechanism of activation of p-type GaN at a relatively low temperature of 500°C in N_2_ ambient, p-type GaN epitaxial wafers without MQWs active region were grown by MOCVD as follows: The epitaxial layers were done by sequentially depositing of a 30-nm-thick low-temperature GaN nucleation layer, a 2.0-μm-thick undoped GaN layer, a 0.6-μm-thick p-type GaN layer, and a 2.0-nm-thick InGaN strained layer on sapphire substrates. X-ray rocking curves showed that the full width at half maximums of the wafers was 299 arcsec for (0002) symmetric plane, indicating a good quality of the p-type GaN layers [21]. A 100-nm-thick ITO film was evaporated on the p-type GaN epitaxial layers by the electron beam evaporator. The sample was then annealed at 500°C in N_2_ ambient for 20 min and finally cleaned in hot ITO acid etching solution for 20 min to remove the ITO film. The sample was marked as sample D. For sample E, the same annealing process was carried out but without depositing ITO film. Subsequently, the two samples were cut into 0.6 cm × 0.6 cm and rinsed in boiling aqua regia for 10 min to remove the native oxides. Finally, Ni/Au dots were evaporated on the surface to form electrical contacts in Vander Pauw geometry for Hall effect measurements [14]. The samples were annealed at 500°C in O_2_ ambient for only 30 s to form ohmic contacts. The short-time annealing process would have fewer influences on the result of the pre-annealing of the samples.

Table 1 shows the hole concentration and Hall effect resistivity of samples D and E at room temperature. Compared to sample E, a significant increase in hole concentration of sample D is observed. Also, it is found that Hall effect resistivity of sample D is only 0.65 Ωcm, while that of sample E is 5.6 Ωcm. These results imply that the capped ITO film can effectively activate p-type GaN at the relatively low annealing temperature in N_2_ ambient. It is known that the presence of oxygen in the annealing environment can result in a positive effect on the activation of p-type GaN. As is noticed that the oxygen may diffuse into the p-type GaN layer during the ITO deposition process; therefore, the possible reason for obtaining highly conductive p-type GaN in sample D was the presence of oxygen either in the ITO layer or p-type GaN layer. To clarify this, sample F experienced the ITO deposition process (the same as sample D) but the ITO layer was removed in acid etching solution before the annealing heat treatment (N_2_; 500°C, 20 min). It is found that Hall effect resistivity of sample F is almost the same as that of the sample E, which proves that the presence of oxygen in the ITO film rather than in p-type GaN helped to activate the p-type GaN. The role of oxygen during annealing process is to enhance the surface reaction for the removal of hydrogen in p-type GaN. For sample D, the oxygen in the ITO film reacted with the hydrogen atoms at the ITO/p-type GaN interface to form H_2_O, which helped to increase the hole concentration during the thermal annealing process.

**Table 1:** **Hole concentration and Hall effect resistivity of the sample D and sample E**

	**Hole concentration (cm** ^**−3**^ **)**	**Hall effect resistivity (Ωcm)**
Sample D	7.5 × 10^17^	0.65
Sample E	1.1 × 10^17^	5.60

The hole concentration of the sample annealed at 500°C in O_2_ ambient was 5.7 × 10^17^ cm^−3^ (not shown in Table 1), which was lower than that of sample D (7.5 × 10^17^ cm^−3^). For the sample D, annealed in N_2_ ambient, the capped ITO film played an important role in providing the oxide to eliminate hydrogen in the p-GaN layer, and N_2_ ambient might suppress the formation of N vacancy-related defects, resulting in higher hole concentration. And this result is consistent with the studies by Wu et al. [22]. Therefore, the p-type GaN was effectively activated at low temperature in N_2_ ambient by employing the ITO film, and the fabrication process for the LED devices was simplified.

**Figure 4:**
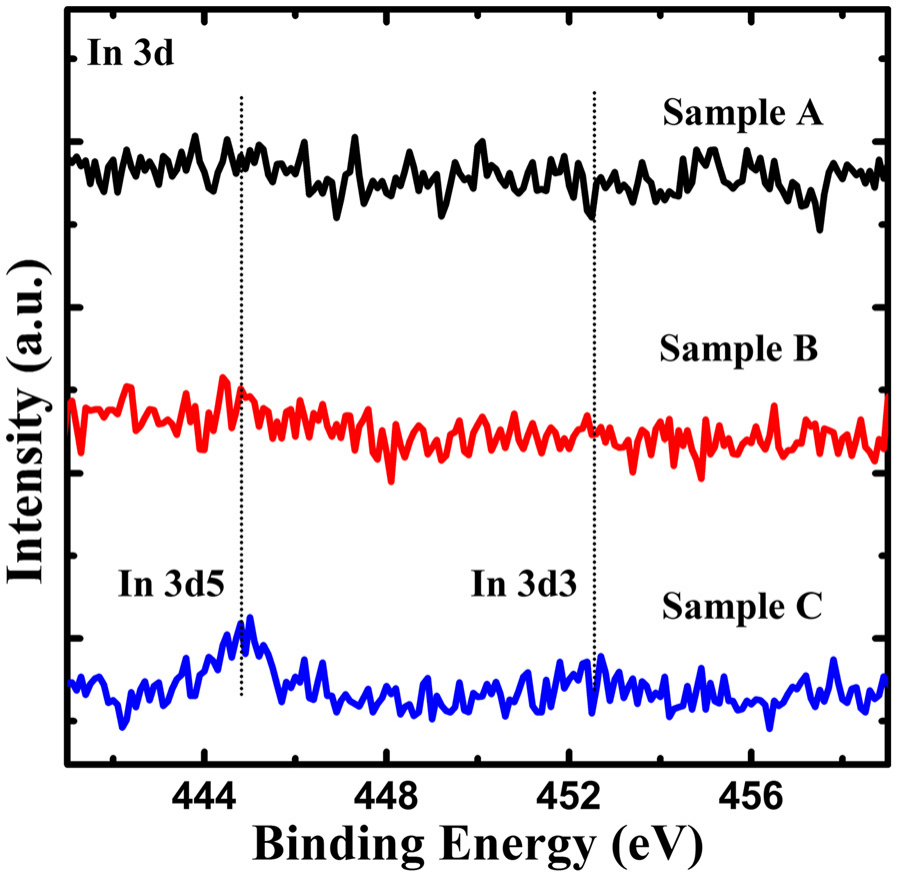
**In 3d core-level XPS spectra for samples A, B, and C at room temperature.**

In order to further describe the improved electrical performance of the LEDs without pre-activation of p-type GaN, the as-grown LED epitaxial samples A, B, and C were prepared for the XPS measurement. The XPS measurement was performed using a Kratos Axis Ultra DLD spectrometer (Kratos Analytical Ltd., Manchester, UK) with Al Kα X-ray (hν = 1486.6 eV) at 12 kV and 120 W. The three samples were rinsed in boiling aqua regia to remove the native oxides before the XPS measurement. Figure 4 shows the In 3d core-level XPS spectra of the surface of the three samples. It is found that the In 3d is almost undetectable on the surface of sample A and sample B. The atomic concentration of In (measured elements including Ga, In, O, and C) for samples A, B, and C is measured to 0.00%, 0.07%, and 0.49%, respectively. Thus, we consider that the In atom in the InGaN strained layer might diffuse into p-type GaN layer during the annealing processes. It is known that the InGaN strained layer can enhance the tunneling transport from the ITO to the p-type GaN layer [16,18]. Therefore, LED sample C without pre-annealing heat treatment could keep a perfect InGaN strained layer between ITO and p-type GaN, which reduced the Schottky barrier height and resulted in forming good ohmic contact. Based on the results of Hall effect and XPS measurements, it can be concluded that the lower forward voltage for the LEDs without pre-activation of p-type GaN was attributed to the highly conductive p-type GaN layer and the formation of good ohmic contact.

## Conclusions

In summary, fabrication of GaN-based LEDs without pre-activation of p-type GaN has been proposed in this work. During the fabrication process, an ITO film was served as the p-type contact layer, and the devices were annealed only once at 500°C in N_2_ ambient for 20 min to increase the transparency of the ITO film as well as activate the p-type GaN. It is found that the LEDs are featured by a lower forward voltage and higher wall-plug efficiency in comparison with the LEDs using pre-activation of p-type GaN. The mechanism of activation of p-type GaN at a relatively low temperature is discussed in detail. The improved performances for the LEDs without pre-activation of p-type GaN are attributed to the highly conductive p-type GaN layer and the formation of good ohmic contact between ITO and p-type GaN. The proposed technique provides a feasible and low cost manufacture for commercialized GaN-based LEDs.

## Abbreviations

LEDs: light-emitting diodes
ITO: indium tin oxide
MOVCD: metal organic chemical vapor deposition
MQWs: multiple-quantum-wells
PL: photoluminescence
WPE: wall-plug efficiency
XPS: x-ray photoelectron spectroscopy

## References

[1] Nakamura S, Mukai T, Senoh M (1994). Candela-class high-brightness InGaN/AlGaN double-heterostructure blue-light-emitting diodes. Appl Phys Lett.

[2] Lee CY, Tzou AJ, Lin BC, Lan YP, Chiu CH, Chi GC (2014). Efficiency improvement of GaN-based ultraviolet light-emitting diodes with reactive plasma deposited AlN nucleation layer on patterned sapphire substrate. Nanoscale Res Lett.

[3] Li SB, Ware M, Wu J, Minor P, Wang ZM, Wu ZM (2012). Polarization induced pn-junction without dopant in graded AlGaN coherently strained on GaN. Appl Phys Lett.

[4] Ohba Y, Hatano A (1994). H-atom incorporation in Mg-doped GaN grown by metalorganic chemical vapor deposition. Jpn J Appl Phys.

[5] Hull BA, Mohney SE, Venugopalan HS, Ramer JC (2000). Influence of oxygen on the activation of p-type GaN. Appl Phys Lett.

[6] Li SB, Zhang T, Wu J, Yang YJ, Wang ZM, Wu ZM (2013). Polarization induced hole doping in graded Al_x_Ga_1-x_N (x = 0.7 ~ 1) layer grown by molecular beam epitaxy. Appl Phys Lett.

[7] Nakamura S, Mukai T, Senoh M, Iwasa N (1992). Thermal annealing effects on p-type Mg-doped GaN films. Jpn J Appl Phys.

[8] Waki I, Fujioka H, Oshima M, Miki H, Fukizawa A (2001). Low-temperature activation of Mg-doped GaN using Ni films. Appl Phys Lett.

[9] Osamura K, Naka S, Murakami Y (1998). Phase separation in InGaN/GaN multiple quantum wells. Appl Phys Lett.

[10] Chuo CC, Lee CM, Nee TE, Chyi JI (2000). Effects of thermal annealing on the luminescence and structural properties of high indium-content InGaN/GaN quantum wells. Appl Phys Let.

[11] Sun L, Weng G-E, Liang M-M, Ying L-Y, Lv X-Q, Zhang J-Y (2014). Influence of p-GaN annealing on the optical and electrical properties of InGaN/GaN MQW LEDs. Phys E.

[12] Kuo C-H, Chang S-J, Su Y-K, Chen J-F, Wu LW, Sheu J-K (2002). InGaN/GaN light emitting diodes activated in O_2_ ambient. IEEE Electron Device Lett.

[13] Koide Y, Maeda T, Kawakami T, Fujita S, Uemura T, Shibata N (1999). Effects of annealing in an oxygen ambient on electrical properties of ohmic contacts to p-type GaN. J Electron Mate.

[14] Sheu JK, Su Y-K, Chi G-C, Koh PL, Jou MJ, Chang CM (1999). High-transparency Ni/Au ohmic contact to p-type GaN. Appl Phys Lett.

[15] Hsu CY, Lan WH, Wu YS (2003). Effect of thermal annealing of Ni/Au ohmic contact on the leakage current of GaN based light emitting diodes. Appl Phys Lett.

[16] Jang J-S, Seong T-Y (2007). Low-resistance and thermally stable indium tin oxide Ohmic contacts on strained p-In_0.15_Ga_0.85_N/p-GaN layer. J Appl Phys.

[17] Horng RH, Wuu DS, Lien YC, Lan WH (2001). Low-resistance and high-transparency Ni/indium tin oxide ohmic contacts to p-type GaN. Appl Phys Lett.

[18] Lee C-L, Lee W-I (2007). Effects of strained InGaN interlayer on contact resistance between p-GaN and indium tin oxide. Appl Phys Lett.

[19] Hung H, Lam KT, Chang SJ, Kuan H, Chen CH, Liaw UH (2007). Effects of thermal annealing on In-induced metastable defects in InGaN films. Mater Sci Semicond Process.

[20] Liu HF, Liu W, Yong AM, Zhang XH, Chua SJ, Chi DZ (2011). Effects of annealing on structural and optical properties of InGaN/GaN multiple quantum wells at emission wavelength of 490 nm. J Appl Phys.

[21] Ke W-C, Lee S-J, Chen S-L, Kao C-Y, Houng W-C (2012). Effects of growth conditions on the acceptor activation of Mg-doped p-GaN. Mater Chem Phys.

[22] Wu LL, Zhao DG, Jiang DS, Chen P, Le LC, Li L (2012). Positive and negative effects of oxygen in thermal annealing of p-type GaN. Semicond Sci Technol.

